# The proximate regulation of prosocial behaviour: towards a conceptual framework for comparative research

**DOI:** 10.1007/s10071-024-01846-w

**Published:** 2024-03-02

**Authors:** Kathrin S. Kopp, Patricia Kanngiesser, Rahel K. Brügger, Moritz M. Daum, Anja Gampe, Moritz Köster, Carel P. van Schaik, Katja Liebal, Judith M. Burkart

**Affiliations:** 1 Comparative Cultural Psychology, Max Planck Institute for Evolutionary Anthropology, Leipzig, Germany; 2 Life Sciences, Institute of Biology, Leipzig University, Leipzig, Germany; 3 Department of Education and Psychology, Freie Universität Berlin, Berlin, Germany; 4 School of Psychology, University of Plymouth, Plymouth, UK; 5 Department of Evolutionary Anthropology, University of Zurich, Zurich, Switzerland; 6 Department of Psychology, University of Zurich, Zurich, Switzerland; 7 Jacobs Center for Productive Youth Development, University of Zurich, Zurich, Switzerland; 8 Institute of Socio-Economics, University of Duisburg-Essen, Duisburg, Germany; 9 Institute of Psychology, University of Regensburg, Regensburg, Germany; 10 Department of Evolutionary Biology & Environmental Studies, University of Zurich, Zurich, Switzerland; 11 Center for the Interdisciplinary Study of Language Evolution, University of Zurich, Zurich, Switzerland

**Keywords:** Prosocial behaviour, Comparative research, Primates, Human children, Sharing, Helping

## Abstract

Humans and many other animal species act in ways that benefit others. Such prosocial behaviour has been studied extensively across a range of disciplines over the last decades, but findings to date have led to conflicting conclusions about prosociality across and even within species. Here, we present a conceptual framework to study the proximate regulation of prosocial behaviour in humans, non-human primates and potentially other animals. We build on psychological definitions of prosociality and spell out three key features that need to be in place for behaviour to count as prosocial: benefitting others, intentionality, and voluntariness. We then apply this framework to review observational and experimental studies on sharing behaviour and targeted helping in human children and non-human primates. We show that behaviours that are usually subsumed under the same terminology (e.g. helping) can differ substantially across and within species and that some of them do not fulfil our criteria for prosociality. Our framework allows for precise mapping of prosocial behaviours when retrospectively evaluating studies and offers guidelines for future comparative work.

## Towards a conceptual framework for comparative research into prosocial behaviour

Acts that favour others, such as helping, comforting, cooperating or sharing, are an essential feature of human behaviour (Batson and Powell 2003). These *prosocial* acts are building blocks of human groups at all levels of organisation (Kaplan et al. 2009). Prosocial behaviour plays a critical role for hunter-gatherers, whose survival depends on mutual aid due to uncertain individual returns of hunting and foraging and occasional episodes of injury or disease (Gurven 2004). In these and other small-scale societies, prosocial acts often occur in the context of partnerships, in which direct and indirect reciprocity play a central role in coordinated subsistence activities, cooperative child rearing, and occasionally warfare (Gurven 2006; Pandit et al. 2017). In large-scale market societies, prosocial behaviour can also include anonymous acts of kindness such as donating money, goods, blood, or even organs to people in need and/or supporting victims of natural disasters or conflicts in other parts of the world (Henrich and Muthukrishna 2021).

Prosocial behaviours are not limited to humans but occur in a broad range of taxa and take place in contexts such as parental and alloparental care, provisioning of food, comforting, protection against predators, territory defence, and conflict resolution (Aureli and de Waal 2000; Clutton-Brock 2009; Jaeggi and Gurven 2013a; Burkart et al. 2017). Typical prosocial behaviours include, for example, infant carrying, food sharing, allogrooming or -preening, alarm calling or consoling (Feistner and McGrew 1989; Ross 2001; Fraser and Bugnyar 2010; Carter and Wilkinson 2013; Schel et al. 2013; Picard et al. 2020). Prosocial behaviours vary in effort and cost for actors: ranging from negligible, such as an orangutan mother bridging a gap between trees to enable her infant to cross (Chappell et al. 2015), to substantial, such as the potential loss of life and limb while defending a group mate against a predator (Boesch 1991; Vogel and Fuentes-Jiménez 2006).

There have been extensive debates about the factors that give rise to prosocial behaviours. From the second half of the twentieth century both psychologists (Rosenhan and White 1967; Darley and Batson 1973; Bar-Tal 1976; Zahn-Waxler et al. 1979) and biologists (Hamilton 1964; Maynard Smith 1964; Trivers 1971; Wilson 1975) started to investigate the *how* and *why* of prosocial behaviour. However, due to their different scientific traditions and paradigms (Okasha 2013; Feigin et al. 2018), the two disciplines have focused on different concepts and research questions. Overall, behavioural biology aims to address all of Tinbergen’s famous four questions (Tinbergen 1963) to understand a particular behaviour (Alcock 2009), with sub-fields such as behavioural ecology often focusing on ultimate explanations and trying to uncover why a particular behaviour evolved and how it benefits (or benefitted) survival and reproduction. Psychologists are often mainly interested in proximate explanations and try to understand what causes and regulates behaviour and how it develops in ontogeny. Failures to acknowledge these different perspectives can lead to (unintentional) misunderstandings and conceptual confusion (Scott-Phillips et al. 2011; Hawley 2014; Pfattheicher et al. 2022), and create obstacles for transdisciplinary work. This applies, for example, to the concepts of ‘altruistic’ (or ‘altruism’) and ‘costs’ in the different fields (West et al. 2007; Pfattheicher et al. 2022).

Psychologists often consider altruistic behaviour a subset of prosocial behaviour (Eisenberg and Miller 1987), and usually conceptualize it as *altruistically motivated* prosocial actions, i.e. driven by concerns for others’ welfare and not by any direct or indirect benefits for the actor (Batson et al. 2008; Eisenberg et al. 2016). Psychologists are usually less concerned about the (immediate) costs of altruistic actions, while other social scientists such as behavioural economists consider only those actions as altruistic that are costly for an actor (e.g. Fehr and Fischbacher 2003). In contrast, altruistic behaviours *in a biological sense* are those that—regardless of their underlying motivation—benefit others and result in *fitness costs* for the actor (Hamilton 1964; West et al. 2007). This implies that altruistic behaviour should be selected against, unless these fitness costs are offset. One likely condition for this is an increase in inclusive fitness, i.e. positive effects on the survival and reproduction of individuals that carry the same gene(s) (Hamilton 1964). Another is *reciprocal altruism* (sometimes also *‘reciprocity’*), a concept emanating from evolutionary biology (Trivers 1971), which refers to costly behaviour that benefits others, but will likely be repaid within the actor’s lifetime. As reciprocation of prosocial actions among non-relatives may be delayed or remain unreciprocated within an actor’s lifetime, some behavioural biologists refer to any costly prosocial behaviour as altruistic to highlight that it can potentially reduce fitness (unless it is reciprocated). Given these conceptual ambiguities across fields (Scott-Phillips et al. 2011; Pfattheicher et al. 2022), we will avoid the terms *altruistic* and *altruism* in this paper.

In the past, due to the diverging interests of biologists and psychologists, empirical work on the proximate regulation of prosocial behaviour was primarily conducted in psychology, particularly in social psychology, personality, and developmental psychology (e.g. Darley and Latané 1968; Eisenberg and Mussen 1989; Zahn-Waxler and Smith 1992). However, since the 1990s, biologists have also become increasingly interested in proximate aspects of prosocial behaviour (e.g. Stander 1992; Boesch 1994; de Waal 1997a; Schino and Aureli 2009). In the twenty-first century, some comparative psychologists and behavioural biologists have joined efforts to study the proximate regulation—particularly the motivational underpinnings—of prosocial behaviour, and, thereby, attempted to transcend the traditional borders of their respective disciplines (e.g. Preston and de Waal 2002; Warneken and Tomasello 2006; Fraser et al. 2008, 2010; Cronin et al. 2009; Drea and Carter 2009; de Waal and Suchak 2010; Greenberg et al. 2010; Massen et al. 2010, 2011; Melis et al. 2011; Berghänel et al. 2011; Yamamoto et al. 2012; Proctor et al. 2013; Burkart et al. 2014, 2017; Jaeggi et al. 2016; Melis and Warneken 2016; Samuni et al. 2018; Picard et al. 2020). These studies have sparked discussions regarding, for example, the cognitive prerequisites for and affective aspects of cooperation, methodological challenges for empirical research on cooperation and prosociality across taxa, and contextual factors influencing the outcome of experiments on prosociality (e.g. Brosnan et al. 2010; Cronin 2012; Marshall-Pescini et al. 2016; Massen et al. 2019; Melis and Raihani 2023). However, comparisons across studies and species may be hampered by considerable variation in the underlying concepts and what counts as prosocial behaviour (Pfattheicher et al. 2022). A *basic* conceptual framework for comparing the proximate regulation of human and non-human animal prosociality would therefore be a valuable tool for anchoring existing studies and guiding future comparative work. Such a framework would need to provide an operational definition of prosocial behaviour that applies to the various forms of prosocial behaviour. This definition would have to be both specific enough to cover human prosociality and broad enough to avoid assumptions that a priori exclude non-human animals. It would also need to work regardless of ultimate factors that may have selected for prosocial behaviour and distinguish prosocial behaviour from inflexible, strongly genetically determined allo-beneficial behaviours in eusocial species (Wilson 1975). The current paper aims to provide such a framework and apply it to selected examples from the empirical literature on human children and non-human primates.

## An operational definition of prosocial behaviour

Here, we define prosocial behaviour as ‘‘voluntary, intentional behaviour that results in benefits for another’’ based on Eisenberg and Miller (Eisenberg and Miller 1987, p. 92). According to this definition, prosocial behaviour (or a prosocial action) is characterised by three features (see also Hawley 2014):

I. the behaviour produces a benefit for one or more individuals other than the actor as a direct result of the actor’s action,
II. the behaviour is intentional, i.e. directed towards the respective outcome for the recipient(s), and not accidental,
III. the behaviour is voluntary, i.e. the actor was not (noticeably) forced to act and would have been able to act otherwise.

This definition has several implications. First, it makes no claims about actions needing to be costly, although most actions will probably entail some cost, even if they only entail opportunity costs. We consider this omission a benefit because both psychologists and biologists sometimes misapply the biological concept of fitness cost (ultimate level) by emphasizing the (immediate) costs of prosocial actions in time or energy (proximate level). Likewise, it allows for actions to have beneficial consequences for the actor later on, for example, by being reciprocated.

Second, we do not specify what motives should underlie prosocial behaviour as such actions may occur due to mixed motives. Possible motives may range from other-regarding motives (i.e. increasing others’ welfare) to self-regarding motives such as feeling competent or obtaining rewards, and may also include strategic considerations like expectations of (direct or indirect) reciprocation (see also Batson 2002; Batson et al. 2008; Eisenberg et al. 2016).

Third, our definition requires individuals to act intentionally, that is, individuals need to have the psychological goal to achieve the outcome. In contrast, we will not consider behaviours as prosocial actions that *accidentally* provide benefits to others, i.e. behaviours that are directed at something else, but produce beneficial results for others as a by-product. This criterion highlights the need for individuals (especially human children and non-human animals) to have the necessary cognitive abilities to understand what actions produce the intended goal (Townsend et al. 2017; Burkart and van Schaik 2020). Ruling out accidental behaviours can be challenging and often controls are needed (comparing a situation with potential beneficiaries being present vs. absent, controls for task understanding or stimulus enhancement, etc.) (Marshall-Pescini et al. 2016).

Fourth, our definition is narrower than other definitions of prosociality (e.g. Batson and Powell 2003), as it does not include intentional, but unsuccessful attempts to benefit another. This conservative feature has the advantage that it relies on observable behaviours with a defined outcome. It does not require researchers to assume actors’ intended goals when actors fail to achieve those goals with their action(s). Consequently, this definition can also be applied to studies of preverbal infants and non-human animals.

Finally, the definition does not prescribe what kinds of behaviour count as prosocial, even though most would intuitively restrict this to affiliative or socio-positive actions. Thus, an attack on an aggressive conspecific or a predator to protect another individual (or the group) or punishment by third parties to enforce group beneficial behaviour also count as prosocial behaviour. Moreover, non-dyadic naturalistic service behaviours such as sentinelling or other forms of anti-predator alarm calling would also be included.

In the next section, we will apply our framework to selected empirical cases of behaviours that are typically referred to as prosocial behaviours. We will primarily focus on non-human primates and human children who have been frequently compared in studies on prosociality and featured prominently in past and current debates (Melis and Warneken 2016). This also reflects our own disciplinary expertise as researchers studying behaviour and cognition in nun-human primates and human children. However, we aim for a broad framework that can be applied to other animal species, including birds and other non-primate mammals, and hope that colleagues who study these species will find the framework helpful.

## Applying the framework to empirical research

Prosocial behaviours have been studied extensively in both human and non-human animals, including, for example, sharing (food, information and objects), targeted helping, consolation, allomaternal care, coalitionary support, and prosocial punishment (Harcourt and de Waal 1992; Aureli and de Waal 2000; Brown et al. 2004; Jensen 2010; Burkart et al. 2014; Melis 2018). Here we will focus on two contexts of prosocial behaviour, food sharing and helping, that have been frequently studied in a range of species (e.g. Feistner and McGrew 1989; Rutte and Taborsky 2007; von Bayern et al. 2007; Ben-Ami Bartal et al. 2011; Bräuer et al. 2013; Carter and Wilkinson 2013; Jaeggi and Gurven 2013a; Massen et al. 2015, 2020; Horn et al. 2016; Lambert et al. 2017; Melis 2018; Liévin-Bazin et al. 2019; Dale et al. 2019; Brucks and von Bayern 2020; Nolte and Call 2021; Laumer et al. 2021; Moscovice et al. 2023). For each context, we will apply our general definition of prosociality to identify what behaviours we consider food sharing and helping, respectively. We will then review both observational and experimental studies with non-human primates and human children to investigate whether they meet our criteria.

### Food sharing

#### Definition

The behavioural patterns, functions, and processes underlying food sharing have been investigated in many primate species (Feistner and McGrew 1989; Jaeggi and van Schaik 2011; Jaeggi and Gurven 2013a). We adapted the definition by Feistner and McGrew (1989) of food sharing as the *tolerated transfer of a defensible item from one motivated individual to another*. We consider a food transfer from one individual to another as sharing if and only if it satisfies all three criteria for prosocial behaviour:

I. the transfer results in a direct benefit (food) for an individual (i.e. recipient) other than the individual possessing/controlling the food,
II. the transfer of food is intended, i.e. it is directed towards the recipient, and not an accidental side effect of some other behaviour, and
III. the transfer is voluntary, i.e. the individual possessing/controlling the food initiates or tolerates the transfer even though it would have been able keep the food.

This definition of food sharing does not presuppose any specific motivation for tolerating the transfer. Moreover, it does not make any assumptions about *how* food is transferred and *who initiates* and/or *performs* the transfer (i.e. whether food is taken by the recipient—*passive transfer*, or given by the possessor—*active transfer*). Correspondingly, the definition neither specifies whether food transfers occur *spontaneously* (unsolicited, *proactive*) or in response to cues/signals of the individual wanting the food (solicited, *reactive*) nor *what* food and *which amount/value* is transferred. In most (and maybe all) animals, control of food will be based on physical possession or proximity, but in humans control can extend beyond physical possession due to mutually recognized ownership rules (Kanngiesser et al. 2020).

#### Observational studies

Observational studies have investigated whether, how and between whom food is transferred under naturalistic or semi-naturalistic conditions (e.g. reviewed in Feistner and McGrew 1989; Brown et al. 2004; Gurven 2004; Jaeggi et al. 2010a), and have often studied mechanisms and functions underlying food transfers in non-human primates and human small-scale societies (e.g. Mitani and Watts 2001; Brown et al. 2005; Jaeggi et al. 2008; Jaeggi and Gurven 2013b; Silk et al. 2013; Crittenden and Zes 2015). Psychological studies with human infants and children, however, have often investigated sharing of resources other than food such as toys.

When food is successfully transferred from one individual to another, the transfer clearly benefits an individual other than the possessor/owner (criterion 1). Accordingly, food-related interactions where no individual is (notably) in possession of the resource would not be classified as *food sharing*: This includes individuals simultaneously feeding on the same food source (co-feeding: de Waal 1989) or an individual picking up discarded leftovers (collect near: de Waal 1989; food retrieval: Nishida and Turner 1996). Some primate species like bonobos (Fruth and Hohmann 2002; Goldstone et al. 2016; Nurmi et al. 2018), chimpanzees (de Waal 1989; Boesch and Boesch 1989; Jaeggi et al. 2010b), capuchins (Rose 1997), or callitrichids (Brown et al. 2004; Guerreiro Martins et al. 2019) have been found to engage in both food sharing and co-feeding. Other species co-feed but have not been observed to share food (e.g. Japanese macaques: Belisle and Chapais 2001; desert baboons: King et al. 2011; rhesus macaques: Dubuc et al. 2012).

In naturally occurring food transfers, it is often difficult to assess whether a transfer was intentional or accidental (criterion 2). Based on Burkart and van Schaik (2020), who recently applied criteria for evaluating intentional communication in primates (Liebal et al. 2014b, chap. 8; Townsend et al. 2017) to food sharing, we suggest that directedness/directed use is the most important and necessary characteristic. Specifically, the possessor must direct their food transfer at a specific individual and might orient their body or gaze towards the recipient.

Active food transfers, in which the possessor directly hands food to the recipient or moves the food within reach of the recipient (Boesch and Boesch 1989), are clearly intentional. For example, in reactive transfers the possessor passes over food in response to a solicitor’s request or more subtle signs of interest. In proactive transfers, the possessor offers food to recipients without recipients (noticeably) signalling their interest in the food (Jaeggi et al. 2010a). While reactive transfers are the predominant form of active food transfers in non-human primates (Jaeggi et al. 2010a; Jaeggi and Gurven 2013a), proactive transfers are rare—except in cooperatively breeding callitrichids (i.e. marmosets and tamarins), which frequently emit food calls to summon immatures and wait with food in their mouth or hand until it is taken (Brown et al. 2004; Burkart and van Schaik 2020).

However, cases of active sharing may exist for which it is difficult to verify whether the transfer was intended. Here we suggest that food transfers can be considered sharing if the possessor is *selective* about who they share with and/or under what circumstances. Consider, for example, the following situation: The possessor is sitting in an elevated position, a piece of fruit falls from their hand to the ground, and an individual below picks it up. More information would be required in this case: Did the possessor take notice of the recipient? Did she respond to a request behaviour? Did she throw the food in the recipient’s direction or drop it seemingly accidentally? In a study with gorillas, food droppings were considered intentional and strategic as they depended on the presence of other individuals and the identity of the possessor and recipient (Iwata 2014).

To assess intendedness, researchers could also investigate whether possessors adjust their sharing behaviour to recipients’ need. For example, adult marmosets, but not squirrel monkeys, are more likely to offer immatures food that is difficult to access (Sehner et al. unpublished data, available at 10.21203/rs.3.rs-2498407/v1). Importantly, their readiness to share with the identical immature decreases when food can be easily accessed.

For passive sharing, recipients are the primary actors. It is therefore often more difficult to determine whether sharing occurred intentionally. However, individuals in control of the food may intentionally refrain from interfering with a food transfer to recipients. This may be challenging for researchers, as they need to evaluate whether the *absence* of an action was intentional. Consequently, some authors do not consider passively tolerated transfers to be food sharing due to the lack of overt actions by possessors (e.g. Liebal and Rossano 2017). However, in most primate species possessors tolerate the taking of defendable food only in exceptional circumstances, and even food-taking attempts by immature offspring are more frequently resisted than accepted (e.g. Nowell and Fletcher 2006; Jaeggi et al. 2008). Rather, individuals’ taking of food is tolerated depending on the partner and the situation (e.g. Boesch and Boesch 1989; Westergaard et al. 1999; Mitani and Watts 2001; Silk et al. 2013; Kopp and Liebal 2016). We therefore suggest considering passive transfers intentional if the possessor can perceive the evident interest of the recipient and *selectively* tolerates the taking of food (de Waal 1989). This will likely require repeated observations of food interactions between possessors and recipients to assess behavioural/situational patterns. The most obvious cases will be those where a higher-ranking individual does not interfere if a lower ranked individual (e.g. an immature) takes food that the higher ranked individual controls (for sharing among adult chimpanzees, see, e.g. Jaeggi et al. 2010b).

Finally, it can be challenging to determine whether a food transfer occurred voluntarily (criterion 3). Dominance hierarchies can offer some help. When a dominant individual tolerates that a subordinate takes food or even hands over food, one can reasonably assume that the behaviour occurred voluntarily as the dominant could have defended the food. The reverse case, however, is more difficult to evaluate because it is less clear whether the subordinate would have been similarly able to defend the food. Again, evaluating whether behaviour occurred selectively may shed further light. For example, did previous instances of refusal result in consequences? It should be noted that we exclude forced transfers (i.e. taking food despite the possessor’s continued resistance, Silk et al. 2013) or stealing (i.e. unexpected, non-preventable snatching of food, de Waal 1989) as they are not voluntary behaviours.

#### Experimental studies

Experimental approaches often explicitly test or, at least, control for voluntariness and intentionality (e.g. Marshall-Pescini et al. 2016), but sometimes fail to fully assess all criteria for food sharing in one study. At a minimum, the experimental setup would need to include (a) an individual in possession/control of food, (b) at least one other food-interested individual, (c) an opportunity for these individuals to interact over food to signal their interest in the food and transfer the food, and (d) an opportunity for the individual in possession/control of the food to choose whether to tolerate a transfer (i.e. to ensure that transfers are voluntary).

There are several food-sharing experiments with non-human primates and human children that fulfil these criteria (de Waal 1997b; Stevens 2004; Sabbatini et al. 2012; Guerreiro Martins et al. 2019; Schino et al. 2021a, b). In studies with capuchins, the focal individual and one or two conspecifics were in test compounds, separated by a mesh, and only the focal had access to food (de Waal 1997b; Sabbatini et al. 2012). The mesh enabled individuals to interact and allowed the possessor to choose whether to approach a conspecific and bring food within their reach. In a food-sharing test with 3- to 5-year-olds in the U.S. (Beier et al. 2019), the experimenter provided the participant with cookies out of a box. She then ‘noticed’ and communicated that there were no cookies left for her, creating a typical opportunity for food sharing. Other developmental studies gave dyads of young children in the U.S., China, and India different amounts of food during snack times and observed cross-cultural differences in how children transferred food (Birch and Billman 1986; Rao and Stewart 1999).

In a frequently used setting to assess prosocial tendencies—in both humans and non-human primates—an actor chooses whether to deliver a (food) reward to a partner or not (Marshall-Pescini et al. 2016). Sometimes, these settings are described as food-sharing tasks (Schaub 1996; Lakshminarayanan and Santos 2008; Brownell et al. 2009; Takimoto et al. 2010), and choosing to provide food for both partner and actor (1/1 option) vs. for just the actor (1/0 option) is considered sharing (Brownell et al. 2009). While setups that give actors full control over the resources (e.g. de Waal 1989; Jaeggi et al. 2010b; Dunfield et al. 2011; Silk et al. 2013) meet our criteria for food sharing, actors in forced-choice studies are in control of the apparatus, but not the food. Specifically, in these tasks the actor does not initially possess the food and therefore cannot potentially keep (and eat) all of it. Once the actor has made a choice, she can only access a pre-allocated portion of food while moving the other (pre-allocated) portion within reach of the recipient. These tasks thus do not measure food transfers from one individual to another and, hence, food sharing according to our definition. On the other hand, a recent study with chimpanzees used a variant of the ultimatum game where the actor could offer between zero to all grapes from their potential possession to a partner (Sánchez-Amaro et al. 2024). Because the actor can freely decide how much (if any) of the food to offer to a partner and keep the rest, this fulfils the criteria for food sharing.

Researchers may need to be cautious when relating results from forced-choice tasks to findings of genuine food sharing settings. To illustrate this: While 18-month-olds from the U.S. did not select a 1/1-option (vs. a 1/0-option) above chance even if an adult-partner explicitly signalled their desire for the snack (they did not ‘share’), 25-month-olds more frequently complied with explicit requests (Brownell et al. 2009; see also Burkart and Rueth 2013). In contrast, when 18- and 24 month-old Canadian children received a box containing snacks, 40% of younger children (and 58% of older children) actively shared their crackers with an adult upon explicit, nonverbal request, but not in a control condition in which the adult had their own crackers (Dunfield et al. 2011). Similarly, chimpanzees, shared food in more naturalistic food sharing tests (e.g. de Waal 1989; Jaeggi et al. 2010b; Silk et al. 2013), but did not prefer the 1/1-option over the 1/0-option in a forced choice task (e.g. Silk et al. 2005; Jensen et al. 2006; Brosnan et al. 2009; Yamamoto and Tanaka 2010).^1^ The latter finding has been used to argue that chimpanzees do not voluntarily share food (Yamamoto and Tanaka 2010). However, findings from food sharing tests and naturally occurring food transfers that match our definition paint a different picture. We suggest that forced-choice tasks are suitable to investigate, for example, other-regarding preferences (if they include necessary controls; Cronin 2012; Marshall-Pescini et al. 2016; Brosnan 2018), but are less appropriate for comparative studies on food sharing.

Finally, some food-sharing studies may address targeted helping rather than sharing. For example, in a recent study an experimenter ‘accidentally’ dropped food items and signalled that he’d like to have them back (‘sharing’) (Barragan et al. 2020). Human children picked up and handed over food items more often in this situation than in a condition where food was thrown away without signs of further interest. Although food was transferred between two individuals, the test was structurally and functionally similar to typical out-of-reach helping tasks (Warneken and Tomasello 2006; Yamamoto et al. 2009). In two other studies, bonobos voluntarily released conspecifics into their enclosure and tolerated subsequent co-feeding (Hare and Kwetuenda 2010; Tan and Hare 2013). Here, no explicit transfer of food occurred and the door opening (that the partner could not have achieved by themselves) provided the partner with access to the remaining food. We therefore suggest that these studies measured targeted helping (see also next section) rather than food sharing in the strict sense.

To summarise, we recommend a conservative approach that only considers those cases as food sharing where food is controlled by an individual, and is either transferred actively or, in case of passive transfers, tolerated selectively.

### Helping behaviour

#### Definition

Helping has been studied widely in humans and non-human primates, including behaviours such as comforting, alloparental care, coalitionary support, handing over items needed for a task, or releasing a conspecific. Here, throughout, we will use the term *helping* in a narrower sense to refer to *targeted* or *instrumental helping* (e.g. Warneken and Tomasello 2006; de Waal 2008; Melis 2018). We define *helping* as *assisting another individual in achieving an action-based goal upon the cognitive appraisal of the specific situation or needs of others* (adapted from de Waal 2008; Svetlova et al. 2010). An act is considered *helping* if and only if it satisfies all three criteria for prosocial behaviour:

I. the action leads to a benefit for the recipient, i.e. produces a result the helpee is trying to achieve,
II. the action is intended to bring about a result based on the helper’s assessment of the situation and/or needs of the recipient, and is not an accidental side effect of some other behaviour, and
III. the action is performed voluntarily, i.e. the actor could have acted otherwise or not at all.

We focus on immediate, action-based goals and include acts like retrieving objects out of others' reach, assisting someone struggling with a practical task, or rescuing someone from an attacker (e.g. Vogel and Fuentes-Jiménez 2006; Warneken and Tomasello 2007; Amati et al. 2008; Chappell et al. 2015). However, the definition excludes general support of another in the pursuit of long-term or abstract goals such as individuals of cooperatively breeding species assisting the mother or parents with raising their offspring (Bales et al. 2000; Kramer 2011). Yet, instances of assisting the immature to reach an immediate, practical goal fit our definition.

#### Observational studies

Observational studies on targeted helping in human children often focus on helping behaviours in everyday situations at home or in kindergartens (Radke-Yarrow and Zahn-Waxler 1976; Dunn and Munn 1986; Svetlova et al. 2010; Brownell and The Early Social Development Research Lab 2016). For example, a study in the U.S. observed 13- to 25 month-old children at home (Dahl 2015, study 2) and considered both handing over objects that were relevant for others’ activities and participation in chores (e.g. sweeping the floor, putting plates on the table) as helping. While children likely assess others’ goals when handing over objects, they may primarily participate in chores to engage with adults (Carpendale et al. 2015). The latter explanation is supported by reports from parents that toddlers, for example, fold but also unfold laundry or load the dishwasher with clean dishes (Hammond 2011). Interestingly, cross-cultural comparisons have shown variation in children’s initiative and involvement in household and everyday chores, with children from indigenous and indigenous-heritage communities in the Americas showing more initiative than North-American children from middle-class backgrounds (Coppens et al. 2016).

Observing helping in non-human primates is often challenging as the situations triggering helping can be difficult to predict. For example, rare, unusual or risky helping events—like rescuing a group member from a predator—are mostly published as case reports (Boesch 1991; Amati et al. 2008; Tokuyama et al. 2012; Matsumoto et al. 2016). More frequently occurring behaviours such as bridging behaviours (i.e. orangutan mothers helping their offspring to cross wide gaps between trees) lend themselves to more systematic studies (Bard 1995; Chappell et al. 2015). While the benefits of supportive actions for the recipient are usually observable, we will again rely on the criteria for intentionality and selectivity, as used in the previous section, to evaluate whether an action was indeed prosocial.

Orangutan mothers bend or sway a tree or make a bridge with their body for their infant to climb along (bridging behaviour: MacKinnon 1974; Rijksen 1978; Bard 1995; van Noordwijk et al. 2009; Chappell et al. 2015), which clearly benefits their offspring. They adjust the amount of assistance to the ability of their infant (Bard 1995) and only help when it cannot cross alone, indicating an assessment of the infant’s needs. Bridging also requires mothers to persevere—often for several minutes—until the infant has crossed the gap (Rijksen 1978). Here, flexible adjustment, selectivity and persistence of behaviour indicate intentionality. Bridging was also observed outside of mother–offspring pairs. For example, Rijksen reported that rehabilitated orangutans occasionally show bridging behaviour for associates and, in one instance, observed a rehabilitated adult female bridge a large gap for a newly introduced juvenile trapped in a tree (Rijksen 1978, pp. 206–207). Moreover, during consortship, females occasionally assist their heavier male consorts cross gaps by pulling trees together (Rijksen 1978, p. 207). These observations strongly suggest that bridging is intentional, voluntary behaviour as orangutans use appropriate measures to fulfil others' needs only when help is needed.

Freeing conspecifics trapped in snares is a difficult task and can result in partial helping or repeated (partial) helping. For example, a male bonobo was caught in a sling attached to a sapling and broke off the sapling by himself, but failed to remove the wire from his hand and the remaining stick, which got caught in a liana (Tokuyama et al. 2012). Group members successfully freed him from the lianas but failed to remove the wire. The supportive behaviour was intended and voluntary (i.e. not forced), yet only partially successful. Moreover, a study with chimpanzees reported sequential helping behaviour (Amati et al. 2008): When a female chimpanzee was caught in the snare, screaming and alarm calling, the alpha male eventually broke off the sapling attached to the snare. When the snare got stuck again and the female could not free herself, he pulled at the stick, but failed to improve her situation. After the female tried to bite through the snare for several minutes, the male finally manipulated the sling with his teeth until it fell off. This sequence shows voluntary, intentional, and appropriate acts to support the female’s goal of removing the snare: he deliberately approached the female, tried various behaviours to free her, adjusted less effective techniques, and continued his effort until he succeeded. Similarly, an observation where a young male chimpanzee assisted an injured mother in carrying her infant and thereby enabled her to keep up with the travelling party, fulfilled all criteria for helping as the assistance was selective and adjusted to the female’s need (Pruetz 2011).

There has been some debate whether rescue behaviour can be considered targeted helping and, in particular, whether it is intentional (Nowbahari and Hollis 2010; Hollis and Nowbahari 2013). However, some forms of rescue behaviour may qualify. For example, during an intergroup encounter between two groups of white-faced capuchins (*Cebus capucinus*), a female and her infant ended up submerged in a river, surrounded and threatened by males from the other group (Vogel and Fuentes-Jiménez 2006). The beta-male from the female’s group responded to her alarm calls, returned, gave a threat call, and then fled while the other group’s males chased after him. The female and her infant and, later, the rescuer, re-joined their group un-harmed. The male’s return to a dangerous situation in response to the female’s alarm calls (while other males did not return) that enabled her to escape with her infant suggests intentional, voluntary behaviour. Furthermore, observations of chimpanzee-leopard encounters (Boesch 1991) report rescue and wound caring behaviours that might qualify as helping (although the author did not label it as such).

#### Experimental studies

Experiments on helping behaviour in human children have focused on motivational, cognitive, social, situational and cultural factors (Svetlova et al. 2010; Köster et al. 2016; Callaghan and Corbit 2018; Köster and Kärtner 2019; Dahl et al. 2022). For example, in early helping experiments (Rheingold 1982), 1.5 to 2.5 year-old U.S. children experienced chores, similar to typical household chores, in the lab and could participate in an adult's activity. Children’s contributions such as holding the shovel for sweeping up bits of paper were considered helping, especially if their assistance occurred unsolicited (but see discussion of toddlers participating in chores above). Frequently, in helping experiments a human experimenter struggles with a practical problem such as reaching for out-of-reach objects or using the wrong means to achieve a goal (Warneken and Tomasello 2006; out-of-reach task replicated, e.g. in Dunfield et al. 2011). Starting in the second year of life, human infants assist experimenters in these situations without being asked to or rewarded but provided little to no assistance in control conditions (without practical problems), indicating that the actions were voluntary and intentional. This has been observed for infants in Brazil, Canada, Germany, India, and Peru (Köster et al. 2016; Callaghan and Corbit 2018; Giner Torréns et al. 2021)—though with some cross-cultural variation in overall helping rates.

In comparable tasks, human-reared juvenile chimpanzees picked up and handed over objects if the experimenter reached for them but did not assist in other scenarios (Warneken and Tomasello 2006). Chimpanzees’ selective response in the out-of-reach tasks may have been based on cognitive assessments of the situation and hence count as helping according to our definition. However, there may be a more parsimonious explanation: As part of positive reinforcement training, captive primates are often rewarded for fetching objects from their enclosure, with caretakers reaching towards objects and calling out to primates. This training effect may explain primates’ behaviour in out-of-reach tasks, even if they were not rewarded during the experiment itself (Drayton and Santos 2014; also discussed in, e.g. Marshall-Pescini et al. 2016; Tennie et al. 2016). Further findings support a more cautious interpretation. In similar test situations, young bonobos did not pass over the object (a stick) and, instead, frequently ‘teased’ the experimenter by, for example, moving it close to their hand and pulling it back (Krupenye et al. 2018). Moreover, capuchin monkeys, who had been trained to retrieve objects for rewards, returned out-of-reach items when rewards were present, regardless of whether experimenters were reaching for objects (Barnes et al. 2008). A follow-up study with the same capuchin monkeys added distractor objects and varied how experimenters referenced target objects to investigate if monkeys were sensitive to humans’ goals (Drayton and Santos 2014). The capuchins retrieved target objects more often than distractors when experimenters reached for them (compared to no reaching). Nevertheless, the authors discuss a simpler explanation than goal representation: Reaching for the target could have simply enhanced monkeys’ attention to the object (Drayton and Santos 2014; Marshall-Pescini et al. 2016).

These studies have all relied on human experimenters in the role of helpee. To create more salient and ecologically valid test situations for non-human primates, other studies have included conspecifics in this role. Commonly, one or more individuals are unable to access desired rewards—usually food—without a conspecific’s assistance. Three settings are often used: (i) the helpee lacks an object/tool to retrieve food, which only the helper can provide (*tool provision*), (ii) the helpee cannot access food unless the helper operates/starts a food delivery device (*food provision*); (iii) the helpee cannot enter a room containing food unless the helper opens a door (*release conspecific*).

In a *tool provision study* both orangutans and—to a lesser extent—chimpanzees voluntarily transferred sticks to a partner, but only orangutans provided sticks selectively when the partner needed them, compared to non-social and no-need control conditions (Liebal et al. 2014a). Orangutans’ tool transfers occurred intentionally according to our criteria, but it is not clear if this was the case for chimpanzees, who may have shared objects rather than helped. In a study with bonobos, female bonobos provided tools to female partners (but not males) more often in the test condition than in a control condition (where the partner had the necessary tool), but did not provide the functional tool selectively (Nolte and Call 2021). Hence, these tool transfers do not clearly meet all criteria of helping. One study, however, demonstrated goal-understanding in chimpanzees in a helping task (Yamamoto et al. 2012): individuals frequently selected the appropriate tool (from a set of objects) when they could see the task the partner had to solve, but otherwise responded randomly.

Studies investigating other-regarding preferences sometimes use token transfer setups that are functionally similar to tool provisioning tasks (Nissen and Crawford 1936; Pelé et al. 2009; Skerry et al. 2011). For instance, brown capuchins voluntarily transferred tokens to a conspecific in an adjacent compound, but also transferred tokens in the recipient’s absence as long as the apparatus was visible (Skerry et al. 2011). Like the study’s authors, we would not view these transfers as helping because they did not occur selectively. Similarly, token-exchange tests with gorillas, chimpanzees, orangutans and bonobos found that only orangutans consistently transferred tokens to recipients upon request (Pelé et al. 2009), but did not consider the token’s value for the partner. Hence, we would not classify those transfers as helping. However, a follow-up study with two of the orangutans demonstrated that—over time—they considered the value of the token for the partner (Dufour et al. 2009).

In a *food provision* study, potential chimpanzee helpers could remove a peg to deliver a reward (food or non-food object) to a conspecific in an opposite compound (Melis et al. 2011). To control for stimulus enhancement, chimpanzees received prior inhibition training involving distractor objects and distractor objects were provided during tests. Chimpanzees released rewards more often when a recipient was present and actively requested more rewards than in non-social or no-request control conditions. Unfortunately, however, requests included pulling a chain connected to the apparatus, which may have simply drawn helpers’ attention to the chain and makes it difficult to assess whether food delivery was an intentional act or an unintended side effect of pulling at the chain. This was investigated in a subsequent study where releasing the peg unlocked or locked a feeding box for recipients (Tennie et al. 2016). Chimpanzees rarely released pegs and releases did not differ between test conditions and control conditions. The authors concluded that releasing behaviour was a by-product of object manipulation triggered by stimulus enhancement.^2^ Engelmann et al. (2019) used a partner-preference paradigm to investigate the impact of relationship quality on helping in human children and chimpanzees. Participants chose between opening a reward box for a “friend” or a “neutral” recipient. Three-year-old German children preferred operating their friend’s box. Instead of a forced partner-choice test, chimpanzees were tested twice with only one partner at a time to whom they could deliver food by removing a peg. On the group level, more chimpanzees delivered food to “friends” than to other partners, but on an individual level, there was only a trend. The authors concluded that this provides evidence for genuine helping (because chimpanzees considered the social relationships) and was not a by-product of stimulus enhancement (for a debate see e.g. Tennie et al. 2016; Melis 2018; Melis et al. 2018). We agree that our criterion for intentionality is met if helpers selectively support particular conspecifics. However, in the absence of controls for stimulus enhancement, lack of non-social control conditions and/or distractor objects, alternative explanations cannot be ruled out.

Similarly, food provisioning in dyadic prosocial choice tests (Silk et al. 2005; Brownell et al. 2009; Cronin et al. 2009; Massen et al. 2010) or group service tests (Burkart and van Schaik 2013; Burkart et al. 2014) may qualify as helping under some conditions. A study with Swiss preschool children (Burkart and Rueth 2013) varied whether actors would receive a reward (1/0 vs. 1/1) or not (0/0 vs. 0/1) and found that children only chose the prosocial option significantly more often (as compared to a non-social control condition) in tests without actor-rewards. An attention analysis revealed that, with the actor’s side baited, they paid no or less attention to the recipient’s side, which accounts for the random choices in the 1/0 vs. 1/1 variant (but see Horn et al. 2018, for a study with older children). Similarly, common marmosets preferred the prosocial option when given a choice between 0/0 vs. 0/1 (Burkart et al. 2007). This selective providing of food can be considered helping according to our criteria. The group service paradigm (Burkart and van Schaik 2013; Burkart et al. 2014) tests individuals in their naturalistic group by baiting a sliding board outside the enclosure that can be pulled within reach by a handle. It is an intuitive task with low cognitive load and hence suitable for comparative research on prosocial behaviour across a broad range of species (Burkart et al. 2014; Horn et al. 2016, 2020; Verspeek et al. 2022; Bhattacharjee et al. 2023). The group service paradigm includes additional control conditions to rule out alternative explanations such as stimulus enhancement (i.e. tapping a non-baited board with a stick) or lack of inhibitory control (i.e. blocking access to the baited board). Helping was operationalized as selective pulling of the baited board in the test phase as compared to the control phase(s). Studies have found that several non-human primate species such as marmosets, tamarins, sakis, siamangs and semi-free ranging Japan macaques (Burkart and van Schaik 2013; Burkart et al. 2014; Bhattacharjee et al. 2023) helped in a group service task. However, due to the naturalistic social setting, it is not always clear to whom the helping is directed in group service tests, especially when several individuals are near the baited board, and would require more detailed analyses, e.g. related to the identity of potential recipients across trials to assess partner preferences and selective food provisioning.

Finally, in *release conspecific* studies, individuals could release a conspecific from a neighbouring enclosure/room into a room containing food. For example, bonobos voluntarily let conspecifics into their own compound containing food by opening a door and did so selectively when another bonobo was present but not when the room was empty (Hare and Kwetuenda 2010; Tan and Hare 2013). When confronted with the choice between releasing a familiar or an unfamiliar conspecific, they predominantly chose the stranger (Tan and Hare 2013). Although both studies purported to investigate food sharing, we suggest that they investigated helping as all criteria for helping—in contrast to sharing—are met: the released individuals given access to desired food, the actions occurred voluntarily (i.e. without external force) and intentionally, as door opening was selective with regard to partner presence and identity, respectively. We emphasis to distinguish between sharing and helping as they may rely on different mechanisms and be differentially regulated.

In sum, experiments that feature conspecifics as helpees and use one of the three settings (tool provision, food provision, release conspecific) are suitable to investigate helping in non-human primates, provided they include appropriate control conditions to rule out alternative explanations (e.g. stimulus enhancement). This also ensures comparability to studies with human children that often use adult or, occasionally, peer partners.

## Conclusion

Prosociality is central, but not limited, to human sociality. Non-human primates and other animals also show prosocial behaviours. While new insights have been gained about the proximate regulation of prosociality in different species over the last decades, progress has also been hampered by a lack of a clear conceptual framework for comparative studies. Here we provide such a framework and show how it can be used to operationalize and compare two frequently studied prosocial behaviours in human children and non-human primates: food sharing and targeted helping. We show that this framework allows for nuanced assessments of prosocial behaviours in these species and we are optimistic that it can also be expanded to other animal species. For example, there is a growing body of prosociality research in birds that uses similar paradigms to the ones discussed here (von Bayern et al. 2007; Massen et al. 2015, 2020; Horn et al. 2016; Lambert et al. 2017; Liévin-Bazin et al. 2019; Brucks and von Bayern 2020; Laumer et al. 2021). Our framework does not only provide a foundation to retrospectively evaluate observational and experimental studies, but can also guide future comparative work.

## Data Availability

Not applicable.

## Footnotes

1 Note though that none of these studies claimed to investigate food sharing.

2 Methodological concerns have been raised about the study, particularly questioning chimpanzee’s understanding of the lock-condition and, therefore, the validity of the results (Melis et al. 2018).
